# Fidelity of the delivery of NHS Health Checks in general practice: an observational study

**DOI:** 10.3399/bjgpopen20X101077

**Published:** 2020-09-23

**Authors:** Ben Paxton, Katie Mills, Juliet A Usher-Smith

**Affiliations:** 1 University of Cambridge School of Clinical Medicine, University of Cambridge, Cambridge, UK; 2 The Primary Care Unit, Department of Public Health and Primary Care, University of Cambridge, Cambridge, UK

**Keywords:** NHS Health Check, fidelity, communication, risk assessment, general practice, Health promotion, Cardiovascular disease, Dementia, Referral and consultation

## Abstract

**Background:**

The NHS Health Check programme aims to reduce the risk of common preventable diseases by providing risk information and behaviour change advice. Failure to deliver the consultation appropriately could undermine its efficacy. To date, to the authors’ knowledge, there are no published data on the fidelity of delivery of NHS Health Checks.

**Aim:**

To assess the fidelity of delivery of NHS Health Checks in general practice.

**Design & setting:**

Fidelity assessment of video and audio recordings of NHS Health Check consultations conducted in four GP practices across the East of England.

**Method:**

A secondary analysis of 38 NHS Health Check consultations, which were video or audio recorded as part of a pilot study of introducing discussions of cancer risk into NHS Health Checks. Using a checklist based on the *NHS Health Check Best Practice Guidance*, fidelity of delivery was assessed as the proportion of key elements completed during the consultations.

**Results:**

The mean number of elements of the NHS Health Check completed across all consultations was 14.5/18 (80.6%), with a range of 10 to 17 (55.6% to 94.4%). The mean fidelity for risk assessment, risk communication, and risk management sections was 8.7/10 (87.0%), 4.1/5 (82.0%), and 1.7/3 (56.7%), respectively. Clinically appropriate lifestyle advice was given in 34/38 consultations. Elements with the lowest fidelity were ethnicity assessment (*n* = 12/38; 31.6%), family history of cardiovascular disease (CVD) assessment (*n* = 25/38; 65.8%), AUDIT-C communication (*n* = 13/38; 34.2%), and dementia risk management (*n* = 6/38; 15.8%).

**Conclusion:**

Although fidelity of delivery was high overall, important elements of the NHS Health Check were being regularly omitted. Opportunities for behaviour change, particularly relating to alcohol consumption and dementia risk management, may be being missed.

## How this fits in

There is disagreement over the value of the NHS Health Check programme. Retrospective analyses of patient records have demonstrated variation in delivery. This is the first study to assess fidelity following observation of recordings of NHS Health Checks. It shows that although fidelity of delivery was high overall, opportunities for behaviour change, particularly relating to alcohol consumption and dementia risk management, may be being missed.

## Introduction

The NHS Health Check programme was introduced in England in 2009 as a primary prevention initiative that aims to prevent CVD through provision of risk information and behaviour change advice.^1^ Since 2013, >6 million people have received an NHS Health Check, making it one of the largest public health prevention programmes in the world.^1^

The NHS Health Check has three sections: risk assessment, risk communication, and risk management. The consultation encompasses a risk assessment for CVD, alongside diabetes, hypertension, and chronic kidney disease (CKD). The risk of developing CVD in the next 10 years should be assessed using the QRISK2 tool.^2^ The QRISK2 score should then be communicated alongside body mass index (BMI), blood pressure, cholesterol, and the AUDIT-C score that describes levels of alcohol consumption.^3^ These results should then form the basis of a discussion about risk management, encompassing lifestyle advice in line with recommendations from the UK Chief Medical Office (to maintain a BMI between 18.5 and 24.9 kg/m^2^, an alcohol consumption rate of ≤14 units/week,^4^ do >150 minutes of moderate intensity exercise/week,^5^ and to not smoke), and medical management options, including prescription of statins and antihypertensive medication.

Although there is strong evidence for each of the risk management options individually, the introduction of the programme has not been without controversy,^6–8^ and the benefits of the programme have been smaller than in early modelling.^9–13^ Alongside an increasing focus on prevention generally, however, both the NHS Long Term Plan^14^ and prevention ‘green paper’, published in July 2019,^15^ cite the importance of NHS Health Checks in the future. Understanding how well the programme is currently being delivered, and whether it is being delivered as intended (that is, the ‘fidelity’),^16^ is therefore important. Fidelity has been shown to affect both patient outcomes^17^ and the conclusions that are drawn in a research context.^18^ It also forms an important part of intervention evaluation^19^ and can highlight areas for improvement.^20^

Previous studies have reported aspects of the delivery.^21,22^ However, these studies have used retrospective analyses of patient records, which may not accurately reflect the content of a consultation.^23^ To date, to the authors’ knowledge, there are no published data reporting fidelity of NHS Health Check delivery based on observation of consultations. This study, therefore, aimed to use video and audio recordings of NHS Health Checks in order to assess the fidelity of delivery against that specified by *NHS Health Check* *Best Practice Guidance*,^1^ and help maximise the impact and inform the future of the programme.

## Method

### Study design and setting

This study was embedded within a pilot study, in which a very brief intervention to share personalised cancer risk information and promote behaviour change was delivered within NHS Health Checks.^24^ Four general practices from across the East of England invited patients to receive a standard NHS Health Check or an extended consultation, during which they also received the very brief cancer intervention. The practices were recruited with the support of the local Clinical Research Network. All four were medium-sized urban practices with list sizes between 8000 and 13 000, and serving a predominantly white population. Based on an overall measure of multiple deprivation experienced by people in the area, three practices were in the two least deprived deciles and the fourth in the fourth least deprived decile. In each general practice, the NHS Health Checks were delivered by a member of the practice nursing team. All used an electronic healthcare record system (SystmOne) that includes a template to complete during the consultation.

### Participants

Each of the four general practices conducted a search of their electronic records for patients who were eligible for an NHS Health Check. This included patients without an existing diagnosis of CVD, CKD, diabetes, hypertension, or hypercholesterolaemia, and not being prescribed statins, or previously been found to have a CVD risk over 20%. Those with a current diagnosis or medical history of cancer or dementia were additionally excluded in this study. Eligible patients were invited by their GP practice to participate in the study. Written informed consent was obtained from each healthcare professional (HCP) and patient before and after the consultation by a member of the study team.

In total, 742 patients were invited to attend an NHS Health Check and participate in the pilot study. Of these, 114 (15.4%) attended without participating in the pilot study, while 41 (5.5%) attended an NHS Health Check within the study, with 34 (4.6%) receiving the cancer risk intervention. One participant withdrew from the study following their consultation, and two did not consent to recording of the consultation. This analysis is, therefore, based on 38 consultations.

### Data collection

Consultations took place between July 2018 and March 2019. Patients completed three questionnaires as part of the wider pilot study: one at baseline prior to the consultation, one immediately afterwards, and one 3 months later. Only data collected at baseline were used in this study. Consultations were video (*n* = 31) or audio (*n* = 7) recorded, dependent on patients’ preference.

### Assessment of fidelity

An initial coding framework based on the *NHS Health Check Best Practice Guidance* was developed to assess fidelity of intervention delivery. One researcher piloted this with four consultations. It was then modified following discussion with the other two researchers. The final coding framework included 18 key elements (Table 1).

**Table 1.** NHS Health Check fidelity checklist based on *NHS Health Check Best Practice Guidance^1^*

**Item**	**Criteria**
**Risk assessment**	
Ethnicity	Asked patient their ethnicity
Height	Measured height
Weight	Measured weight
Blood pressure	Measured blood pressure
Pulse rhythm	Palpated pulse and verbalised comment regarding pulse
Cholesterol	Evidence of cholesterol measurement taken
Family history of CVD	Asked patient about any family history of cardiovascular disease
Alcohol usage	Asked patient about alcohol consumption per week
Smoking history	Asked patient about smoking status
Weekly physical activity	Asked patient number of hours of physical activity completed per week
**Risk communication**	
QRISK2 score	Told patient their QRISK2 percentage score
Blood pressure	Told patient their blood pressure
BMI	Told patient their BMI
Cholesterol	Told patient their cholesterol
AUDIT-C	Told patient their AUDIT-C score
**Risk management**	
Lifestyle advice	Suggested, or encouraged the patient to suggest, specific lifestyle changes for risk reduction
Clinically appropriate lifestyle advice	As for 'lifestyle advice', but where lifestyle advice is given which relates to at least one lifestyle factor for which the patient was outside of the recommended limits
Dementia risk information	Told patient the key message that the risk factors for CVD and dementia are the same

BMI = body mass index. CVD = cardiovascular disease.

One researcher then assessed fidelity of all consultations by watching the video recordings or listening to the audio recordings. Each element of the NHS Health Check was assessed as either completed (‘yes’) or not completed (‘no’). Where elements were assessed as not completed, this was subsequently checked by searching for relevant phrases within the written transcripts. A second researcher assessed five consultations (13.2%) to check intercoder reliability.

### Analysis

For each consultation, fidelity was expressed as the proportion of elements of the NHS Health Check completed. Lifestyle advice was categorised based on the lifestyle risk factor it most closely related to: weight loss (including dietary changes), reducing alcohol consumption, increasing physical exercise, or smoking cessation. To identify whether clinically appropriate lifestyle advice was given, the authors determined whether a patient was outside of the UK Chief Medical Officer’s recommended ranges, and received lifestyle advice relating to at least one of these lifestyle factors. Patients who were within the recommended range for all four key lifestyle factors, but still received lifestyle advice, were included as having received clinically appropriate lifestyle advice.

## Results

The characteristics of participants are shown in Table 2. The mean age was 52.2 years (standard deviation [SD] = 9.3); 68.4% (*n* = 26) were female; the majority were of white ethnicity (89.5%, *n* = 34); 50.0% (*n* = 19) reported a family history of CVD in a first-degree relative; and 18.4% (*n* = 7) had a QRISK2 score >10%.

**Table 2.** Patient characteristics

**Characteristic**		***n* (%)^a^**
**Mean a** **ge, years** **(** **range, SD)**		52.2(40 to 73, 9.3)
**Sex**	Female	26 (68.4)
	Male	12 (31.6)
**Ethnicity**	White	34 (89.5)
	Mixed/multiple ethnic group	0 (0.0)
	Asian/Asian British	1 (2.6)
	Black/African/Caribbean/Black British	3 (7.9)
**Family history of CVD in a first-degree relative**		19 (50.0)
**BMI >25**		26 (68.4)
**Weekly moderate physical exercise <2.5 hours**		14 (36.8)
**Weekly alcohol consumption >14 units**		7 (18.4)
**Current smoker**		2 (5.3)
**QRISK2 >10%**		7 (18.4)
**Education level**	University	18 (47.4)
	Secondary	19 (50.0)
	Primary	1 (2.6)
**Deprivation quintile** ^b^	1	4 (10.5)
	2	14 (36.8)
	3	11 (28.9)
	4	9 (23.7)
	5	0 (0.0)

^a^Unless otherwise stated. ^b^Index of Multiple Deprivation quintile: 1 = least deprived, 5 = most deprived. BMI = body mass index. CVD = cardiovascular disease. SD = standard deviation.

The intervention was delivered by seven HCPs: three practice nurses and four healthcare assistants (HCAs).

Intercoder reliability for fidelity assessment was 90%. The mean total length of the NHS Health Check consultation was 14 minutes 38 seconds (SD = 5 minutes 58 seconds; confidence interval = 12 minutes 44 seconds to 16 minutes 31 seconds). There were no substantial differences in fidelity of delivery between the standard NHS Health Checks (*n* = 7) and the NHS Health Check plus cancer intervention (*n* = 31) (results not shown).

The overall fidelity assessment broken down by HCP is shown in Table 3. The mean number of elements completed across all consultations was 14.5/18 (80.6%), with a range of 10 to 17 (55.6% to 94.4%). The range of the mean number of elements completed by each HCP was 13.6 to 16 out of 18.

**Table 3.** Overall fidelity assessment for each HCP

**HCP**	**Role**	**Consultations, *n***	**Elements (*N* = 18)** **completed, mean (%)**	**Elements (*N* = 18) completed, range (%)**
**A**	HCA	5	14.0 (77.8)	11 to 16 (61.1 to 88.9)
**B**	Practice nurse	2	14.5 (80.6)	14 to 15 (77.8 to 83.3)
**C**	Practice nurse	2	16.0 (88.9)	15 to 17 (83.3 to 94.4)
**D**	HCA	6	15.0 (83.3)	15 to 15 (83.3 to 83.3)
**E**	HCA	18	14.1 (78.4)	12 to 17 (66.7 to 94.4)
**F**	Practice nurse	3	15.3 (85.2)	14 to 16 (77.8 to 88.9)
**G**	HCA	2	14.0 (77.8)	14 to 14 (77.8 to 77.8)

HCA = healthcare assistant. HCP = healthcare professional.

The mean fidelity for risk assessment, risk communication, and risk management sections was 8.7/10 (87.0%), 4.1/5 (82.0%), and 1.7/3 (56.7%), respectively. Table 4 shows the breakdown of fidelity for each of the elements within these sections. Elements with the lowest fidelity were ethnicity assessment (*n* = 12/38; 31.6%), family history of CVD assessment (*n* = 25/38; 65.8%), AUDIT-C communication (*n* = 13/38; 34.2%) and dementia risk management (*n* = 6/38; 15.8%) .

**Table 4.** Fidelity by NHS Health Check element

	**Element of NHS Health Check**	**Consultations** **completed, *n* (%)**
**Risk assessment**	Ethnicity	12 (31.6)
	Height	38 (100)
	Weight	38 (100)
	Blood pressure	38 (100)
	Pulse rhythm	29 (76.3)
	Cholesterol	36 (94.7)
	Family history of CVD	25 (65.8)
	Alcohol consumption	38 (100)
	Smoking	38 (100)
	Physical activity	38 (100)
**Risk communication**	QRISK2	37 (97.4)
	Blood pressure	36 (94.7)
	BMI	35 (92.1)
	Cholesterol	34 (89.5)
	AUDIT-C	13 (34.2)
**Risk management**	Lifestyle advice	34 (89.5)
	Clinically appropriate lifestyle advice	34 (89.5)
	Dementia	6 (15.8)

BMI = body mass index. CVD = cardiovascular disease.

For dementia risk management, patients were made aware ‘*that the risk factors for cardiovascular disease are the same as those for dementia*’ in only 15.8% (*n* = 6/38) of consultations. Of those aged 65 to 74 years (*n* = 3), who are required to also be signposted to further support where appropriate, 100% were offered further support.

Lifestyle advice was given in 89.5% (*n* = 34/38) consultations. Of the patients who were not already meeting the recommendations for ≥1 of the lifestyle factors, 87.5% (*n* = 28/32) received clinically appropriate lifestyle advice for at least one of the factors while 12.5% (*n* = 4/32) patients received no lifestyle advice (Table 5). Alcohol consumption advice was the lifestyle advice given least often for patients where it would have been clinically appropriate.

**Table 5.** Clinically appropriate lifestyle advice

		Of participants with lifestyle factor outside recommended range, number receiving:
**Lifestyle Factor**	**Participants outside of UK Chief Medical Officer’s recommended range, *n* (%)**	**Risk assessment communicated,^a^*n* (%)**	**Clinically appropriate lifestyle advice, *n* (%)**
Overweight	26 (68.4)	24 (92.3)	22 (84.6)
Exercise	14 (36.8)	No relevant risk score	9 (64.3)
Alcohol	7 (18.4)	5 (71.4)	4 (57.1)
Smoking	2 (5.3)	No relevant risk score	2 (100)

BMI = body mass index.

a Risk scores communicated: weight = BMI; alcohol consumption = AUDIT-C.

## Discussion

### Summary

To the authors' knowledge, this is the first study to report fidelity of NHS Health Check delivery based on the assessment of recorded consultations. This study has shown that although overall fidelity of delivery was high among the sample of consultations included in this study, important elements of the NHS Health Check were regularly omitted.

In particular, this study shows that less than a third of patients were directly asked their ethnicity as part of the risk assessment, despite ethnicity being included as a risk factor within the QRISK2 calculation, and screening guidelines for hypertension and CKD, and CVD risk, being known to vary between ethnic groups.^25^ The AUDIT-C score was also communicated in only 34.2% of consultations, compared with BMI and cholesterol, which were communicated to 92.1% and 89.5% of patients, respectively. Dementia risk messaging was completed in only a small minority of consultations (15.8%). This was despite all the HCPs in this study appearing to make use of templates that would have included these elements within the consultations. Such a difference between template guidance and activity has been reported previously.^26^ In this context, the low number of consultations in which the results of the AUDIT-C assessment and dementia risk were discussed may reflect either a lack of familiarity among HCPs with the *N* *HS Health Check* *Best Practice* *G* *uidance* and/or template, particularly for the dementia risk assessment that was only included in 2017; HCPs not feeling comfortable raising dementia with younger individuals or alcohol consumption more generally; or HCPs not considering those elements to be of benefit.

In contrast, this study found that overall fidelity of delivery of clinically appropriate lifestyle advice was high (*n* = 34/38 consultations; 89.5%), in keeping with the requirement that patients should receive clinically appropriate lifestyle advice ‘*regardless of their risk scor* *e*’.^1^

### Strengths and limitations

The main strength of this study was the use of audio and video recordings to directly observe fidelity of delivery of the NHS Health Check. This approach, however, meant that only activity within the consultation itself was captured, and this assessment did not take account of pre-existing data on practice records or referrals that were made after the consultation. For this reason, this was not included in the fidelity assessment elements, which were likely to have been completed outside of the consultation itself, such as CKD risk assessment. However, this may still have affected some of the elements within the risk assessment section. It may, for example, explain the low percentage of consultations in which ethnicity was asked if data on ethnicity were already in the practice record.

The inclusion of cancer risk information at the end of the standard NHS Health Check as part of the pilot study for 31 patients, and the potential impact of this on normal delivery of the NHS Health Check, is another key limitation. Audio/video recording of consultations may also have changed the way in which HCPs delivered the NHS Health Check. The authors suggest, however, that since the emphasis of the pilot study, and the training delivered to the healthcare practitioners in relation to that, was on the cancer risk intervention, and delivery of the standard NHS Health Check was not mentioned, this effect would not have been significant. Although the numbers were small, this study also saw no substantial differences between delivery of the NHS Health Checks between the standard NHS Health Checks and those with the additional cancer risk information included.

While the small sample size of patients and HCPs enabled recording of the consultations and more in-depth assessment of delivery, the small numbers are also a limitation. Moreover, the study took place in four GP practices in one geographical area of the UK and, therefore, the authors of this study are unable to comment on regional variations. The participants were also predominantly of white ethnicity, well-educated, and many already met the lifestyle recommendations. This meant the authors are also unable to comment on whether HCPs might approach NHS Health Check consultations differently with different groups of individuals.

Finally, this study focused only on the fidelity of delivery of NHS Health Checks and did not assess the other domains of fidelity (design, training, receipt, and enactment).^16^ Additionally, only the adherence of the delivery was assessed, and not the quality.

### Comparison with existing literature

While this is the first study, to the authors’ knowledge, to assess fidelity following observation of recording of NHS Health Checks, other studies have reported fidelity using retrospective analyses of patient records. Using data from a bespoke audit system, Baker *et al* ^21^ reported the variation in NHS Health Check completion across 83 GP practices in Gloucestershire. Consistent with this study's findings, they showed that alcohol assessment was comparatively low, with only 53.9% having a documented alcohol assessment. Documented assessments of smoking (83.2%), physical activity (87.8%), and cholesterol (75.7%) were higher, and closer to levels found in this study. Documented lifestyle advice was substantially lower though with only 31.1%, 66.9%, and 44.2% reported to have been given clinically appropriate advice or a referral relating to diet, smoking, and exercise, respectively.

Using data from electronic medical records from 1066 NHS Health Checks delivered across 13 practices in North West England, Krska *et al* ^22^ found similarly high levels of recorded risk assessment (>90%) and lifestyle advice given (80.6%). The levels of clinically appropriate lifestyle advice given for alcohol (92.9%) were higher than in the present study. The differences between these studies and the present study may be the result of regional differences or changes in practice over time, and may also reflect known inconsistencies between electronic documentation of consultations and direct observation.^23^

Previous studies have also reported reluctance among HCPs to discuss alcohol with patients. Reasons for this include concerns about the effect on the relationship with the patient, the stigma associated with alcohol, and lack of training.^27,28^ All these may contribute to the low rate of communication of the results of the AUDIT-C assessment seen within this study.

### Implications for practice and research

For GPs and other HCPs involved in the delivery of NHS Health Checks, this study's findings particularly highlight the potential missed opportunities relating to dementia risk management and identifying those who may benefit from alcohol interventions. Dementia and alcohol training resources have been developed by Public Health England, and existing templates within electronic health records may be useful to improve the consistency of delivery of these elements. The use of risk reports^29^ may also improve communication. Further research with those delivering the NHS Health Checks is needed, however, to identify why these elements are being omitted, and if further training or resources are indeed the solution or if additional changes are needed. Until then, GPs in practices offering NHS Health Checks should review the delivery within their own practices to ensure that key opportunities for behaviour change, particularly those relating to dementia and alcohol, are not being missed.
